# Cardiovascular events in patients under age fifty with early findings of elevated lipid and glucose levels – The AMORIS study

**DOI:** 10.1371/journal.pone.0201972

**Published:** 2018-08-23

**Authors:** Torbjörn Ivert, Håkan Malmström, Niklas Hammar, Axel C. Carlsson, Per E. Wändell, Martin J. Holzmann, Ingmar Jungner, Johan Ärnlöv, Göran Walldius

**Affiliations:** 1 Department of Molecular Medicine and Surgery, Karolinska Institutet and Heart and Vascular Theme, Karolinska University Hospital, Stockholm, Sweden; 2 Unit of Epidemiology, Institute of Environmental Medicine, Karolinska Institutet, Stockholm, Sweden; 3 Biostatistics, Data Management and Medical Writing, Research & Development, Swedish Orphan Biovitrum (Sobi), Stockholm, Sweden; 4 Medical Evidence and Observational Research, Global Medical Affairs, AstraZeneca, Mölndal, Sweden; 5 Division of Family Medicine and Primary Care, Department of Neurobiology, Care Sciences and Society, Karolinska Institutet, Huddinge, Sweden; 6 Department of Medical Sciences, Uppsala University Hospital, Uppsala, Sweden; 7 Department of Emergency Medicine, Karolinska University Hospital, Huddinge, Sweden; 8 Department of Internal Medicine, Karolinska Institutet, Solna, Sweden; 9 School of Health and Social Studies, Dalarna University, Falun, Sweden; University College London, UNITED KINGDOM

## Abstract

**Background:**

The long-term trajectories of lipid and glucose levels in subjects who experience a major cardiovascular (CV) event at a young age has not been well studied. Our objective was to investigate lipid, lipoprotein, apolipoprotein (apo), and glucose levels in individuals experiencing a CV event before 50 years of age.

**Methods and findings:**

A first CV event [non-fatal myocardial infarction (MI), coronary revascularisation, or CV related death] before age 50 was recorded in 2,939 (cumulative incidence 1.2% in males and 0.3% in females) of 361,353 individuals included in the prospective Swedish AMORIS (Apolipoprotein-related MOrtality RISk) study with health examinations 1985–1996 and follow-up through 2011. In a nested case-control analysis, cases with a CV event were matched to randomly selected controls. Population risk factor trajectories were calculated up to 20 years prior to an event. Total cholesterol (TC), triglyceride (TG), and glucose levels were higher in cases than in controls as early as 20 years prior to the event with differences increasing over time. Low density lipoprotein, apoB, and the apoB/apoA-1 ratio were higher and increased over time, while HDL and apoA-1 were lower in cases compared to controls. The odds ratio was 2.5 (95% confidence interval 1.6–3.7) for TC ≥5 mmol/L and TG ≥1.7 mmol/L in cases versus controls. The adjusted population-attributable fractions including lipids, glucose, diabetes, smoking, hypertension, and obesity indicated that about 50% of CV events before age 50 may be associated with elevated lipid and glucose levels.

**Conclusions:**

Elevated TC, TG, LDL, apoB, and glucose levels and high apoB/apo A-1 ratio documented two decades before a CV event in subjects younger than 50 years may account for about half of CV events before age 50, which calls for early recognition and possibly treatment of modifiable CV risk factors in young individuals.

## Introduction

Dyslipidaemia, smoking, hypertension, and diabetes are known risk factors for atherosclerosis and its complications, primarily myocardial infarction (MI) and stroke [1–7]. Atherosclerotic changes have been observed at young ages [8, 9],but clinical manifestations are uncommon in individuals younger than 50 years, although risk factors may be present [4, 5, 10–14]. High total cholesterol (TC) and glucose levels have been associated with an increased risk of cardiovascular (CV) disease in young persons [7, 10]. Asymptomatic men with multiple risk factors have an increased risk of death from CV causes [15]. The risk of a future CV event can be estimated from information on sex, age, smoking, TC, and blood pressure [1]. In recent decades, the prevalence of cardiovascular disease, diabetes, and, especially, obesity in young individuals and women has increased worldwide [1, 16]. Therefore early identification of CV risk factors is of importance from a primary prevention perspective. The long-term pattern of CV risk factors in individuals who experience a major CV event at a young age has not been extensively studied.

The goal of this nested case control study was to review and evaluate trajectories of lipid, lipoprotein, apolipoprotein, and glucose levels in the years preceding a CV event in individuals younger than 50 years.

## Methods

### Design and study population

The study was based on the Apolipoprotein-related MOrtality RISK (AMORIS) Cohort. The AMORIS study was designed to study apolipoprotein (apo) B (atherogenic) and apoA-1 (athero-protective) levels relative to future CV disease [17–19] and includes 812,073 subjects chiefly from the greater Stockholm area who underwent health examinations with laboratory testing through occupational health screening or primary care from 1985–1996.

For the present study, we selected from the AMORIS cohort subjects younger than 50 years who had measurement of total cholesterol (TC), triglycerides (TG), and glucose at the initial health examination (baseline) (n = 361,353 individuals; 46% females). No subject was hospitalized at the time of the baseline health examination or had experienced MI or coronary revascularization prior to this date. Information on low-density lipoprotein cholesterol (LDL), high-density lipoprotein cholesterol (HDL), and apoB and apoA-1 as well as traditional CV risk factors including tobacco smoking and hypertension was obtained in sub-sets of patients. To analyse long-term trajectories of CV risk factors we conducted a nested case control study within the cohort.

All data were anonymous before any analyses. Informed consent was not required. The study complied with the Declaration of Helsinki and was approved by the Regional Ethical Review Board in Stockholm (2010/1047-31/1, 2011/1406-32].

### Case identification and follow-up

For the identification of a first CV event before age 50 years, records of all subjects were reviewed through 2011 in the Swedish National Patient Registry and the National Cause of Death Registry by linkage using a unique personal identification number assigned to all individuals living in Sweden. A first CV event was defined as a non-fatal MI, coronary revascularisation [coronary artery bypass grafting (CABG) or percutaneous coronary intervention (PCI)], or CV death. The mean period from initial data collection to event was nine years, and the maximum was 27 years. The Swedish National Patient Registry includes information on inpatient care regionally from 1964 and nationally from 1987. The National Cause of Death Registry includes all deaths recorded in Sweden along with information on the cause of death. Information regarding coronary revascularization was obtained from the Swedish Web System for Enhancement and Development of Evidence-based Care in Heart Disease Evaluated According to Recommended Therapies (SWEDEHEART) [www.swedeheart.se]. No individual experiencing a previous MI or revascularisation before the index examination was included in the analyses.

### Patient characteristics

To obtain information on patient characteristics beyond the laboratory data, the AMORIS cohort was linked to several additional registers and research cohorts described in detail with references in an AMORIS cohort paper [19]. In brief, information on socioeconomic class was obtained from mandatory Swedish national censuses from 1970 to 1990. Information on diabetes, history of smoking, blood pressure, and self-reported hypertension was obtained from several sources including research cohorts at the Karolinska Institutet (the Work, Lipids, and Fibrinogen study, the Cohort of Swedish Men study, the Swedish Mammography Cohort, the cohort of 60-year-old subjects in Stockholm, the Sollentuna Primary Prevention Study and for women undergoing pregnancy also from the national Swedish Medical Birth Register) [19]. Information on smoking and blood pressure was obtained from the SWEDHEART registry for many of the individuals experiencing a CV event, hence providing more data for CV cases than for subjects with no CV diagnosis. Care was taken to, as much as possible, implement similar definitions of patient characteristics derived from different sources in the analyses of this study. Information on dispensed medication was available for all subjects by the National Prescribed Drug Register beginning in July 2005.

### Blood sampling and laboratory analyses

Analyses were conducted on fresh blood serum samples (55% fasting) at CALAB Medical Laboratories, Stockholm, Sweden. Total cholesterol and TG were determined by enzyme techniques, and LDL, HDL, and apolipoproteins were measured as previously described [18, 19]. Glucose levels were analysed with the glucose oxidase/peroxidase technique, using automated multichannel analysers [AutoChemist-PRISMA® (New Clinicon, Stockholm, Sweden) and Technicon DAX® TM 96 (Technicon Instruments Corp., Tarrytown, NY, USA)]. Creatinine levels were assessed with the non-kinetic alkaline picrate method (Jaffé), using AutoChemist-PRISMA from 1985 through 1992 and, from 1993 through 1996, a DAX-96 analyser. The coefficient of variation was <3% for all laboratory tests. The glomerular filtration rate was estimated using the Chronic Kidney Disease Epidemiology Collaboration formula.

### Definitions

Cardiovascular deaths were classified according to the International Classification of Diseases (ICD)-9 as 390–459 and, from 1997, as ICD-10 codes I00–I99. Acute myocardial infarction was defined as ICD-9 code 410 or ICD-10 code I21. Percutaneous coronary intervention and CABG included all interventions reported to the SWEDEHEART registry. Body mass index was calculated as weight in kg divided by the square of height in meters, with obesity defined as BMI ≥ 30. Hypertension was identified as either >140/90 mm Hg, self-reported hypertension, or a dispensed anti-hypertensive drug recorded in the National Prescribed Drug Registry. Socioeconomic status was classified as manual or non-manual occupation and by level of education (≤9 years, 9–12 years, or >12 years). High serum glucose level was defined as a fasting glucose level of ≥7 mmol/L (126 mg/dL), a random glucose level of ≥11.1 mmol/L (200 mg/dL), or a diagnosis of diabetes registered in the National Diabetes Register [20]. To reflect the relative contribution of TG to TC as a risk factor, combined dyslipidaemia was defined as Fredrickson classification [21]—*Type IIa* with TC ≥ 5 mmol/L (193 mg/dl) and TG < 1.7 mmol/L (150 mg/dl); *Type IIb* with TC ≥ 5 mmol/L and TG level ≥ 1.7 mmol/L; or as *Type IV* with TC < 5 mmol/L and TG ≥ 1.7 mmol/L.

### Statistical methods

We used the entire cohort to calculate the cumulative incidence of a first CV event before age 50. We estimated sex- and age-specific incidence rates (number of events per person-years at risk) for subjects exhibiting a combination of total serum cholesterol ≥6.0 mmol/L, triglycerides ≥1.40 mmol/L, and glucose ≥5.6 mmol, as well as incidence rate ratios with 95% confidence intervals.

Population trajectories of risk factors and association of a first CV event with risk factors were analysed using a nested case-control approach. Cases included all subjects experiencing a CV event as defined above (non-fatal MI, coronary intervention, or CV death) before age 50 during the study period. Incidence density sampling was used to randomly select five control subjects per case from the cohort population at risk of a new CV event [22]. Controls were matched to cases according to age (five-year cohorts), sex, and calendar year of the event. We generated trajectories of mean values of risk factors in cases and controls separately, starting 25 years prior to diagnosis of the case or selection as a control subject. These trajectories constitute a comparison between cases and controls at given time points and do not represent repeat measurements in the same individual. The trajectories are based on a single baseline assessment per subject. They do not represent individual development over time, but the mean values of cases and controls each year prior to diagnosis of the case or selection as a control subject. This type of trajectory can be designated a population trajectory.

Population trajectories for TC, TG, glucose, lipoprotein, and apolipoprotein mean values with 95% confidence intervals (CI) were calculated stratified by years preceding the CV event or selection as control. The geometric mean was used for TG because of the skewed distribution. We did not perform statistical tests to compare differences in mean values of cases and controls but calculated 95% confidence intervals for the means of cases and controls. Non-overlapping confidence intervals indicated significant difference between cases and controls. Logistic regression was used to calculate odds ratios (OR) with 95% CI for risk of a CV event. Cut-off levels were set to ≥ 5 mmol/L and ≥1.7 mmol/L for hypercholesterolaemia and hypertriglyceridaemia, respectively [1]. SAS software 9.3 TS Level 1M0 (SAS Institute Inc., Cary, NC, USA) was used for data programming.

## Results

### Entire study group

We identified 2,939 subjects with a first CV event before age 50. The events occurred four times more often in males (1.2%; 2,422/197,095) than in females (0.3%; 517/177,333). Few events occurred before age 40, with the majority (61%) at 45–49 years.

The estimated incidence rate of a CV event before age 50 years in subjects 40–49 years of age was 47/10,000 person-years in males and 15/10,000 person-years in females with initial measurements of serum cholesterol ≥6.0 mmol/L, triglycerides ≥1.40 mmol/L, and glucose ≥5.6 mmol/L (Table 1). This represented an incidence rate ratio of 2.6 in males and 3.0 in females compared to subjects with levels below these values.

**Table 1:** Incidence rate and incidence rate ratio with 95% confidence interval for an adverse cardiac event before age 50 among 361,353 men and women stratified to three age groups with total cholesterol ≥6.0 mmol/L, triglycerides ≥1.40 mmol/L, and glucose ≥5.6 mmol/L (Exposed) and those with lower levels (unexposed).

		Exposed (n = 8,467)			Unexposed (n = 352,886)			
Sex	Age at initial testing (years)	Number of events	Person years	Incidence rate /10,000 years	Number of events	Person years	Incidence rate /10,000 years	Incidence rate ratio (95% CI)
Male	20–29	18	5,122	35	461	999,480	5	7.0 (4.4–11.2)
Male	30–39	104	21,996	47	986	921,445	11	4.3 (3.5–5.2)
Male	40–49	109	23,187	47	726	405,435	18	2.6 (2.1–3.2)
Female	20–29	1	1,769	6	119	900,186	1	6.0 (0.8–42.9)
Female	30–39	8	3,315	24	207	733,296	3	8.0 (3.9–16.2)
Female	40–49	7	4,624	15	163	353,052	5	3.0 (1.4–6.4)

### Nested-case control analyses

The nested case-control study with matching to five controls was performed in 2,925 CV cases (99.5%), with 14 cases matched to fewer controls due to lack of available appropriate referent subjects. A total of 14,660 controls were included. Cardiovascular cases and controls were well balanced by design with respect to age, sex, and calendar year of the event (Table 2). The mean time from the initial blood sampling to a CV event or selection as control was nine years. All subjects exhibited normal renal function. Smoking, diabetes, hypertension, manual work, and fewer than nine years of education were more common, while body mass index, TC, TG, LDL, apoB, and the ratio apoB/apoA-1 and glucose levels were higher, among CV cases than controls. HDL and apoA-1 were lower in cases than in controls.

**Table 2:** Characteristics of subjects experiencing an adverse cardiovascular event before age 50 and controls.

Variable	Cases (n = 2,939)	Controls (n = 14,660)	*P*-value
Years since blood sampling $\bar{x}$ (*SD*)	9.25 (6.15)	9.03 (6.18)	
Age ^a^ (years) $\bar{x}$ (*SD*)	45.4 (4.22)	45.0 (4.51)	
Female (%)	18	18	
History of smoking (%)	62	25	<0.001
Diabetes/High glucose level^b^ (%)	13	3	<0.001
Hypertension (%)	35	5	<0.001
eGFR <60 mL/min/1.73 m^2^ (%)	0	0	0.61
Body mass index (kg/m^2^) $\bar{x}$ (*SD*)	26.7 (4.82)	24.2 (3.84)	<0.001
Body mass index ≥30 (kg/m^2^) (%)	21	6	<0.001
Manual work (%)	53	40	<0.001
Non-manual work (%)	41	54	<0.001
Unclassified (%)	6	5	0.5910
Education ≤9 years (%)	27	19	<0.001
Education 9–12 years (%)	47	45	0.0950
Education >12 years (%)	20	34	<0.001
Education Unclassified (%)	6	2	<0.001
TC (mmol/L) ^c^ $\bar{x}$ (*SD*)	6.06 (1.36)	5.30 (1.09)	<0.001
TG (mmol/L) $\bar{x}$ (*SD*)	1.91 (1.51)	1.34 (1.04)	<0. 001
Glucose (mmol/L) $\bar{x}$ (*SD*)	5.29 (2.09)	4.85 (0.94)	<0.001
LDL (mmol/L) $\bar{x}$ (*SD*)	4.24 (1.29)	3.47 (1.01)	<0.001
Non-HDL (mmol/L) $\bar{x}$ (*SD*)	5.06 (1.51)	4.06 (1.31)	<0. 001
HDL (mmol/L) $\bar{x}$ (*SD*)	1.23 (0.41)	1.43 (0.40)	<0.001
Apolipoprotein B (mmol/L) $\bar{x}$ (*SD*)	1.49 (0.43)	1.23 (0.36)	<0. 001
Apolipoprotein A-1 (mmol/L) $\bar{x}$ (*SD*)	1.29 (0.22)	1.37 (0.21)	<0.001
ApoB/ApoA-I ratio $\bar{x}$ (*SD*)	1.20 (0.50)	0.93 (0.31)	<0.001
Fredrickson Classification			
IIa (TC ≥ 5, TG <1.7) (%)	39	41	0.10
IIb (TC ≥ 5, TG ≥1.7) (%)	41	19	<0.001
IV (TC < 5, TG ≥1.7) (%)	4	5	0.024
TC < 5, TG <1.7 (%)	16	36	<0.001

TC, total cholesterol; TG, triglycerides; LDL, low density lipoprotein cholesterol; HDL, high density lipoprotein cholesterol; eGFR, estimated glomerular filtration rate

^a^ Age at first event or selection as control subject

^b^ Diabetes mellitus or fasting glucose ≥7 mmol/L or any glucose ≥11 mmol/L

^c^ Geometric means.

Available data (Cases/Controls); Smoking (1,374/1,485), Diabetes (2,766/14,066); Hypertension (972/6,228); Body mass index (1,169/3758); Apolipoprotein (1,076/4,340)

### Trajectories

**Fig 1:**
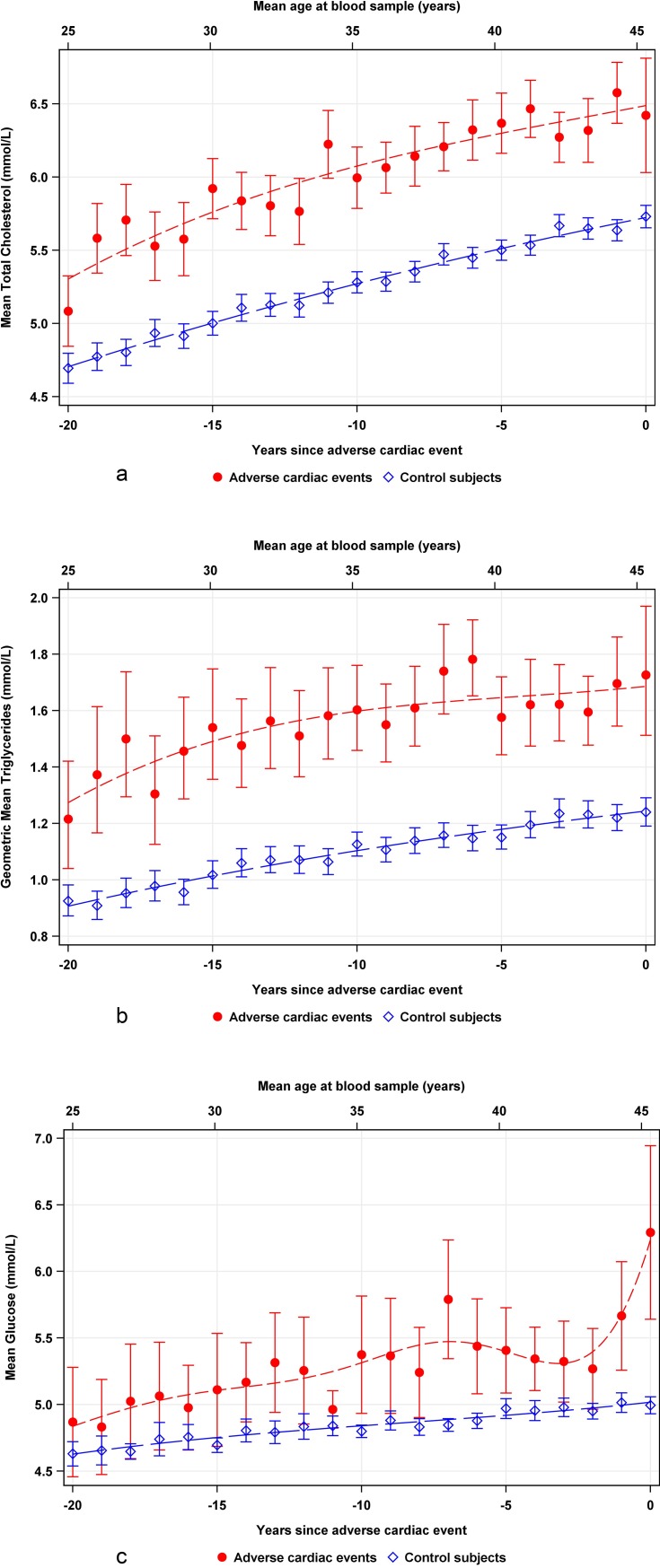
Weighted scatterplot smoothed curves of mean values with 95% confidence interval (CI) for **(a)** total cholesterol, **(b)** triglycerides, and **(c)** glucose in 2939 cases (red line) and 14660 controls (blue line) obtained during 20 years preceding a CV event. Clinical reference levels are indicated on the y-axis. Mean age is given at the top of each graph, and years before a cardiac event is indicated on lower x-axis.

Total cholesterol and TG levels were found significantly higher in CV cases than in controls 20 years prior to a CV event. Total cholesterol, TG, and glucose levels increased continuously and almost in parallel with time closer to the event in both cases and controls (Fig 1A–1C). The slope for glucose was steeper among cases than controls a few years before the CV event.

**Fig 2:**
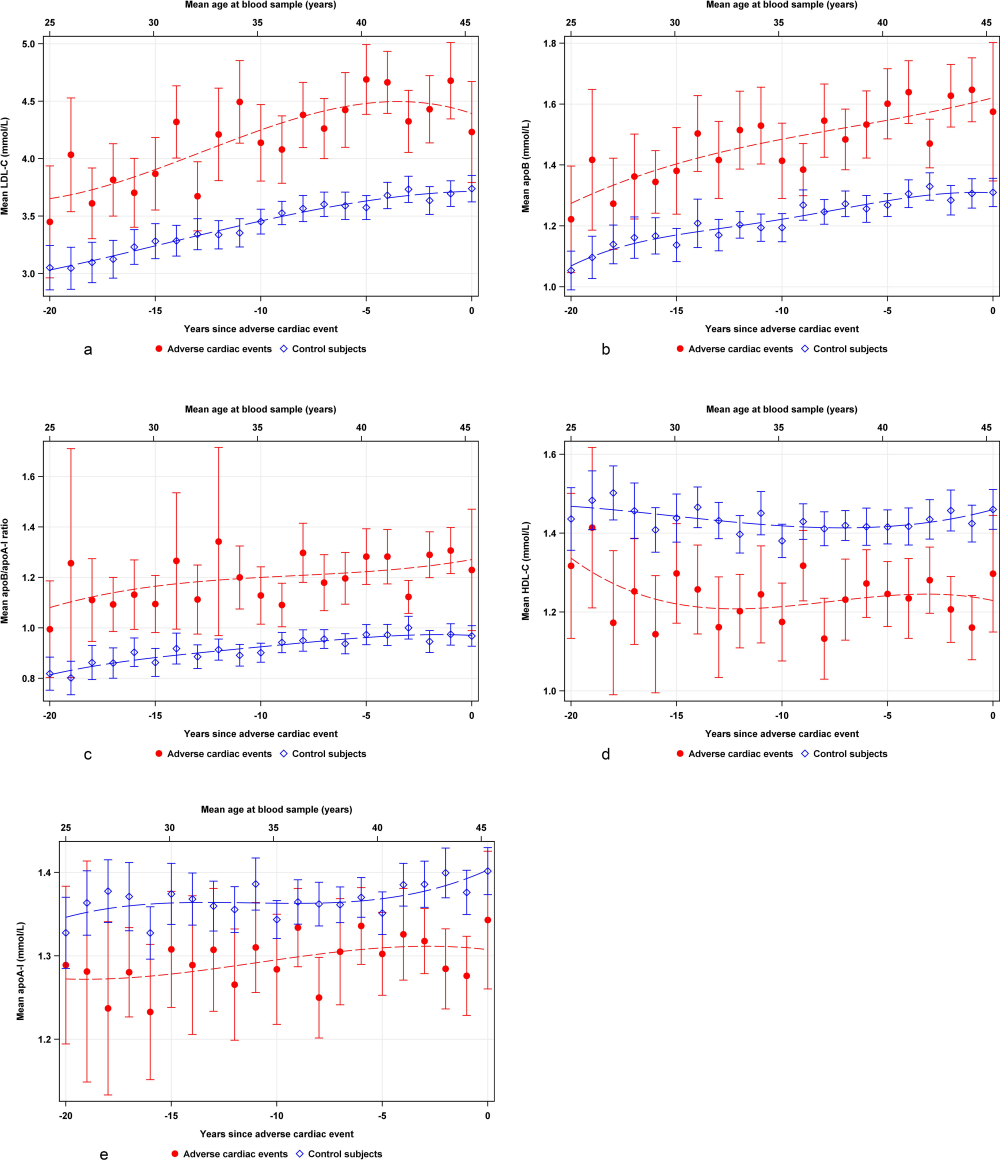
Weighted scatterplot smoothed curves of mean values with 95% confidence interval (CI) for **(a)** low density lipoprotein cholesterol (LDL) in 1164 cases (red line), and 4988 controls (blue line), **(b)** apolipoproteins B (988 cases, red line; 3927 controls blue line), **(c)** apolipoproteins B/A-1 (940 cases, redline; 3596 controls, blue line) ratio **(d)** high density lipoprotein cholesterol (HDL) (1144 cases; red line, 4963 controls, blue line) **(e)** apolipoproteins A-1 (1076 cases, red line; 4340 controls, blue line) obtained during 20 years preceding a CV event. Clinical reference levels are indicated on the y-axis. Mean age is given at the top of each graph, and years before a cardiac event is indicated on lower x-axis.

Low density lipoprotein levels were higher in cases than in controls 20 years before the event and increased in parallel with controls but decreased closer to the event (Fig 2A). The ApoB values and the apoB/apoA-1 ratio were higher in cases than in controls and increased during 20 years before the event (Fig 2B and 2C). High density lipoprotein and apoA-1 remained almost stable up to the event in both cases and controls (Fig 2D and 2E).

### Risk of a CV event

Complete information of target variables was available for 628 cases and 859 controls (S1 Table). High TC and TG levels, hyperglycaemia or diabetes mellitus, smoking, hypertension, and obesity were all significantly associated with an increased incidence of a CV event before age 50 (Table 3). The multivariable adjusted population attributable risk (PAR) suggested that ~50% of CV events before age 50 may be associated with elevated TC, TG, and hyperglycaemia. High PAR values were also observed for smoking, hypertension, and obesity (Table 3).

**Table 3:** Odds ratios (OR) and population attributable risk (PAR) with 95% confidence intervals (CI) for an adverse cardiovascular event before age 50 calculated for cases and controls from available data.

Variable	Cases(628)^a^ (%)	Controls(859)^b^ (%)	OR (95% CI)	Adjusted ^c^ OR (95% CI)	%PAR (95% CI)	Adjusted ^c^ %PAR (95%CI)
High TC (≥5 mmol/L)	72.1	42.9	2.0 (1.5–2.6)	1.5 (1.1–2.1)	36.4 (24.1–46.7)	24.5 (7.2–38.5)
High TG (≥1.7 mmol/L)	39.9	10.9	2.9 (2.1–4.0)	1.7 (1.2–2.4)	26.1 (17.7–33.6)	15.9 (4.6–25.8)
Diabetes/High glucose^d^	15.6	2.4	6.4 (3.7–11.3)	3.3 (1.8–5.9)	13.2 (5.5–20.3)	10.8 (2.8–18.1)
History of smoking	64.0	25.7	3.1 (2.4–4.1)	2.8 (2.1–3.7)	43.6 (34.8–51.2)	41.1 (31.5–49.4)
Hypertension	33.3	7.6	4.0 (2.8–5.7)	2.9 (2.0–4.2)	25.0 (17.2–32.0)	21.6 (13.2–29.2)
BMI ≥ 30 (kg/m^2^)	24.5	3.6	6.7 (4.2–10.8)	4.1 (2.4–6.8)	20.9 (13.3–27.8)	18.5 (10.4–25.8)

BMI, body mass index; TC, total cholesterol; TG, triglycerides.

^a^ Data available in 628/2939 cases (22%)

^b^Data available in 859/14,660 controls (6%)

^c^Adjusted for variables in Table 2

^d^Diabetes mellitus or fasting glucose ≥7 mmol/L or any glucose ≥11 mmol/L.

Combined high TC and TG (*Type IIb* hyperlipidaemia) (adjusted OR 2.5, 95% CI 1.6–3.7) and isolated hypertriglyceridaemia (type IV) were associated with a more than doubled risk of a CV event before age 50 compared to controls. Isolated hypercholesterolaemia (Type IIa) showed lower risk (Table 4).

**Table 4:** Odds ratios (OR) with 95% confidence intervals (CI) for a cardiovascular event before age 50 years in relation to hyperlipidaemia, calculated for cases and controls with available data.

Blood lipid levels according to Fredrickson[18]	Cases (n = 628) (%)	Controls (n = 859) (%)	OR (95% CI)	Adjusted ^a^ OR (95% CI)
TC < 5 and TG <1.7 (mmol/L)	24.0	55.1	1.0	1.0
Type IIa, TC ≥ 5, TG<1.7 (mmol/L)	36.1	34.0	1.7 (1.3–2.3)	1.6 (1.2–2.3)
Type IIb, TC ≥ 5, TG≥1.7 (mmol/L)	36.0	8.9	3.8 (2.6–5.5)	2.5 (1.6–3.7)
Type IV, TC <5, TG = >1.7	3.8	2.1	3.9 (1.8–8.4)	2.6 (1.1–6.3)

TC, total cholesterol; TG, triglycerides.

^a^ Adjusted for all variables presented in Table 2

In subjects with multiple risk factors *Type IIb* hyperlipidaemia, history of smoking and hypertension was associated with an adusted risk of a CV event nine-fold that of non-smokers without hypertension and with TC < 5 mmol/L and TG <1.7 mmol/L (OR 9.1, 95% CI 3.9–21.2) (Table 5)

**Table 5:** Odds ratios (OR) of obesity, high glucose level, and combinations of smoking, hypertension, and type IIb hyperlipidaemia for a cardiovascular event before age 50 years in cases and controls.

Variable	Cases (n = 628) (%)	Controls(n = 859) (%)	OR (95% CI)	Adjusted OR (95% CI)
BMI = > 30 (kg/m^2^)	24.5	3.6	6.7 (4.1–11.0)	4.5 (2.7–7.5)
High glucose level^a^	15.6	2.4	6.3 (3. 6–11.2)	3.4 (1.8–6.2)
Type IIb hyperlipidaemia	6.6	4.0	3.57 (2.0–6.5)	3.0 (1.6–5.5)
Smoking	26.9	19.3	3.59 (2.5–5.1)	3.6 (2.5–5.2)
Hypertension	7.7	4.4	3.30 (1.9–5.8)	2.5 (1.4–4.6)
Smoking and hypertension	12.2	1.9	14.6 (7.6–28.1)	10.6 (5.4–20.7)
Type IIb+Hypertension	4.5	0.4	19.7 (5.2–74.6)	14.8 (3.4–63.9)
Type IIb and smoking	16.0	3.6	8.1 (4.7–13.8)	6.8 (3.9–11.7)
Type IIb+smoking+hypertension	9.1	0.9	16.4 (7.0–38.3)	9.1 (3.9–21.2)

BMI, body mass index.

^a^fasting glucose level of ≥7 mmol/L, any glucose ≥11 mmol/L, or diagnosis of diabetes

Fredrickson *Type IIb* hyperlipidaemia, high glucose level, smoking, obesity, and low socioeconomic status were more common among the 1,315 subjects (45%) who had undergone coronary revascularization than among the 1,624 subjects (55%) experiencing non-fatal MI or CV-associated death (Table 6). Those subjects showed a longer time from the baseline measurement to event than those with MI/CV death (11 versus 6 years)

**Table 6:** Characteristics of subjects undergoing PCI or CABG and those with MI or death from cardiovascular cause before age 50.

Variable	All cases n = 2939		PCI/CABG n = 1315 (45%)		MI/Death n = 1624 (55%)		Difference PCI/CABG and MI/death (p Value)
Years to event (median, (range))	8 (4–14)		11 (7–16)		6 (3–11)		<0.001
Age years (mean (SD))	45.4 (4.21)		45.9 (3.4)		44.9 (4.7)		<0.001
Fredrickson Classification		(%)		(%)		(%)	
IIa (TC ≥ 5, TG <1.7)	1132/2918	38.8	491/1304	37.7	641/1614	39.7	0.07
IIb (TC ≥ 5, TG ≥1.7)	1198/2918	41.1	570/1304	43.7	628/1614	38.9	<0.001
IV (TC < 5, TG ≥1.7)	105/2918	3.6	47/1304	3.6	58/1614	3.6	nc
TC < 5, TG <1.7	483/2918	16.6	196/1304	15.0	287/1614	17.8	0.003
Female	517/2939	17.6	185/1315	14.1	332/1315	20.4	<0.001
High glucose level^a^	348/2766	12.6	197/1221	16.1	151/1545	9.8	<0.001
History of smoking	863/1374	62.8	628/967	64.9	235/407	57.7	<0.001
BMI ≥30 (kg/m^2^)	251/1169	21.5	172/700	24.6	79/469	16.8	<0.001
SES/Low	1569/2939	53.4	731/1315	55.6	838/1624	51.6	0.002
Hypertension	342/972	35.2	266/759	35.0	76/213	35.7	0.35
GFR <60 mL/min/1.73 m^2^	24/2939	0.82	6/1315	0.46	18/1624	1.1	<0.001

BMI, body mass index; CABG, coronary artery bypass grafting, CVD, cardiovascular disease; MI, myocardial infarction; GFR, glomerular filtration rate; nc, not calculated; PCI, percutaneous coronary intervention; SES, socioeconomic status; TC, total cholesterol; TG, triglycerides.

^a^ Diabetes mellitus or fasting glucose ≥7 mmol/L or any glucose ≥11 mmol/L

## Discussion

To the best of our knowledge, this study based on the AMORIS cohort is the largest prospective study evaluating associations of major CV risk factors with a CV event before age 50 years. We found that TC, TG, LDL, apoB, the apoB/apoA-1 ratio, and glucose levels were higher in cases experiencing a CV event than among matched controls as early as 20 years before the event, i.e. at age 30 or younger. Trajectories showed that all potentially atherogenic fractions TC, TG, LDL, apoB, and the apoB/apoA-1 ratio increased and remained higher over time, whereas the protective HDL and apoA-1 remained lower in cases than in controls up to the event. These accumulated differences in lipids, lipoproteins, and apolipoproteins for many years may be strong drivers of atherosclerosis risk resulting in CV events.

At a young age, TC and TG were higher in those experiencing a CV event than in controls, albeit below diagnostic cut-off levels commonly used in adult populations. In agreement with previous studies, we found smoking, obesity, hypertension, and low socioeconomic status to be associated with adverse CV events in young individuals [5, 12, 23]. Information on smoking and hypertension was more frequently available for CV cases than for controls.

Glucose levels were higher in CV cases than in controls, and increased more substantially closer to the CV event. This may indicate early development of hyperglycaemia. Our findings of a significant contribution of high glucose levels, i.e. signs of pre-diabetes (impaired fasting glucose) and manifestations of metabolic syndrome or diabetes confirmed findings of an earlier study [24]. In individuals hospitalized for MI, impaired glucose metabolism is a common finding, with less than 35% showing normal glucose tolerance [25]. In a previous study, we found elevation of lipids, lipoproteins, and the apoB/apoA-1 ratio to be present about 15 years before diagnosis of type 2 diabetes [26].

Our data are in accordance with epidemiological and genetic evidence supporting elevated TG as a major risk factor for CV disease and mortality [27]. We found the combination of smoking, hypertension, and *Type IIb* hyperlipidaemia according to the Fredrickson classification to be associated with a nine-fold risk of a CV event before age 50 years compared to controls. The increased risk of a CV event associated with *Type IIb* and *Type IV* dyslipidaemia reaffirms the need for early diagnosis and management of lipid levels, particularly when combined with other risk factors.

The value of health check-ups and laboratory screening for young individuals is often questioned, as in a Cochrane review of 152,435 asymptomatic participants [28]. However, when high TC and TG levels are recognized early in life, effective treatment can reduce CV risk [29]. Thus, patients with familial hypercholesterolaemia or combined *Type IIb* and *Type III* dyslipidaemias are candidates for pharmaceutical intervention at a young age, especially if the case of hereditary dyslipidaemia [29]. Clearly, attention to young individuals presenting multiple conventional risk factors, including metabolic factors, is warranted to detect significant risk and to offer appropriate preventive therapy. This may be particularly important, since lipid and glucose disorders, including type 2 diabetes, contribute to increase in worldwide prevalence of CV disease in young men and women. The estimated population-attributable fractions suggest that approximately 50% of CV events before age 50 could be prevented if elevated TC, TG, and glucose levels were normalized. These results are consistent with the INTERHEART study findings of a 60% population-attributable for myocardial infarction associated with risk high apoB/apoA-1 ratio in combination with diabetes in individuals of mean age 58 years from 52 countries world-wide [19, 30, 31]. Our findings of high LDL, high apoB, and, especially, high apoB/apoA-1 ratio indicate that imbalance between atherogenic (LDL and apoB) and athero-protective (HDL and apoA-1) lipoproteins is a major risk factor for severe CV disease not only in elderly but also in early life. The apoB/apoA-1 ratio has emerged in international studies as a solid and important predictor of risk of CV events [16,18, 31].

The strengths of this study include the investigation of a large cohort of young individuals with records reviewed up to 20 years, enabling assessment of lipids, lipoproteins, apolipoproteins, and glucose levels at least two decades preceding a CV event before age 50. A large proportion of the blood samples were from occupational health screenings in healthy subjects, with analyses of fresh blood samples conducted by a single laboratory. In the interpretation of the trajectories, it is important to note that these represent differences between cases and controls at time of measurement of the biomarkers relative to time of diagnosis. The trajectories do not represent a time series of data for a single individual. Similar population trajectories were previously used in an AMORIS study on development of type 2 diabetes [26].

The timing of the blood sampling relative to the CV event and inclusion of control subjects is of critical importance to interpretation of the results of this study. We had access to the dates of both the examinations and the CV events. The National Patient, National Cause of Death, and SWEDEHEART registries cover dates, CV diagnoses, and interventions with a high degree of completeness. The diagnostic quality in the national registers used to identify cases in this study is high, and any misclassification of disease is unlikely to substantially influence our findings. Our present findings are based on a cohort that has been shown to be representative of the employed population of greater Stockholm County according to the 1990 census with regard to social class, country of birth, and marital status [19]. The AMORIS cohort comprised about 30% of the population of Stockholm County during the inclusion period. Since a large segment of the cohort was included via routine health screenings in the occupational setting, there was a higher proportion of employed subjects in the AMORIS cohort compared to the general population. Consequently, the AMORIS cohort is associated with a healthy worker effect, and the standardized mortality ratio was 0.86 compared to the general population in Stockholm County for the study period [19]. This may have led to an underestimate of the absolute rate of CV events under age 50, but is less likely to have biased the internal validity of the comparison of risk factor levels in CV cases and controls.

An important limitation of this study was that information on apolipoproteins, smoking, hypertension, and obesity were, due to screening procedures, not available for all subjects, which restricts the completeness of multivariable analyses. Notably, smoking habits and hypertension were more commonly reported in individuals who had been in contact with the health care system, especially those who had suffered a CV event.

In addition, we did not have access to several other factors that may affect the likelihood of developing CV disease, including family history, diet, alcohol consumption, abdominal obesity, physical activity, working hours, environmental factors, and level of psychosocial stress. However, since the analyses of population trajectories was basically descriptive, lack of adjustment for other major risk factors in these analyses may not be a major limitation but needs to be kept in mind in the interpretation.

Total cholesterol, TG, LDL, apoB, apoB/apoA-1 ratio and glucose levels were higher in cases compared to controls up to two decades before a CV event occurring before age 50. The differences between levels in cases and controls increased over time up to the event and may account for about half of CV events before age 50. This provides support for early identification and possibly treatment of modifiable CV risk factors in young individuals.

## Supporting information

S1 Table Characteristics of subjects with complete variables experiencing an adverse cardiovascular event before age 50 and controls. (PDF)
