# Supplementary material for: Cardiovascular events in patients under age fifty with early findings of elevated lipid and glucose levels – The AMORIS study
Source: PLoS One. 2018 Aug 23;13(8):e0201972. doi: 10.1371/journal.pone.0201972 (PMC6107147; doi:10.1371/journal.pone.0201972)
Supplement: S1 Table — (PDF) [file pone.0201972.s001.pdf]

S1 Table Characteristics of subjects with complete variables experiencing an adverse cardiovascular event before age 50 and controls

Variable	Cases (n=628)	Controls (n=859)
No. years since blood sampling $\bar{x}$ (*SD*)	15.4 (5.4)	13,2 (5,8)
Age [a] (years) $\bar{x}$ (*SD*)	46.0 (3.3)	43.2 (5.1)
Female (%)	18	79
History of smoking (%)	63	22
Diabetes/High glucose level[b] (%)	15	2
Hypertension (%)	32	7
eGFR <60 mL/min/1.73 m^2 (%)	0	0
Body mass index (kg/m^2) $\bar{x}$ (*SD*)	27,2 (4.8)	23.2 (3.4)
Body mass index ≥30 (kg/m^2) (%)	24	3
Socioeconomic status		
Manual workers (%)	34	44
Non-manual employees (%)	57	48
Unclassified (%)	7	6
Years of education		
≤9 years (%)	24	13
9-12 years (%)	52	53
>12 years (%)	21	32
Unclassified (%)	3	2
TC (mmol/L) $\bar{x}$ (*SD*)	5,7 (1.2)	4,9 (0,9)
TG (mmol/L)[c] $\bar{x}$ (*SD*)	1,5 (1,3)	0,9 (0,7)
Glucose (mmol/L) $\bar{x}$ (*SD*)	5, (1,4)	4,6 (0,7)
LDL (mmol/L) $\bar{x}$ (*SD*)	3,8 (1,0)	3,0 (0,9)
Non-HDL (mmol/L) $\bar{x}$ (*SD*)	4,5 (1.3)	3,5 (0,9)
HDL (mmol/L) $\bar{x}$ (*SD*)	1.6 (0.4)	1.6 (0.4)
Apolipoprotein A-1 (mmol/L) $\bar{x}$ (*SD*)	1.27 (0.2)	1.4 (0.2)
Apolipoprotein B (mmol/L) $\bar{x}$ (*SD*)	1.3 (0.4)	1.1 (0.3)
ApoB/ApoA-I ratio $\bar{x}$ (*SD*)	1.1 (0.6)	0.8 (0.2)
	(%)[b]	(%)[b]
Fredrickson Classification		
IIa (TC ≥ 5, TG <1.7) (%)	36	33
IIb (TC ≥ 5, TG ≥1.7) (%)	35	8
IV (TC < 5, TG ≥1.7) (%)	3	2
TC < 5, TG <1.7 (%)	26	57

TC, total cholesterol; TG, triglycerides; LDL, low density lipoprotein cholesterol; HDL, high density lipoprotein cholesterol; eGFR, estimated glomerular filtration rate

[a] Age at first event or selection as control subject; [b]Diabetes mellitus or fasting glucose ≥7 mmol/L or any glucose ≥11 mmol/L; [c] Geometric means.
